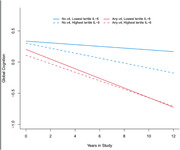# Prospective Associations of Interleukin‐6 and APOE 4 Allele with Cognitive Decline in Biracial Community‐dwelling Older Adults: the Chicago Health and Aging Project (CHAP)

**DOI:** 10.1002/alz.089243

**Published:** 2025-01-09

**Authors:** Ted K.S. Ng, Todd Beck, Pankaja Desai, Klodian Dhana, Robert S. Wilson, Denis A Evans, Kumar B Rajan

**Affiliations:** ^1^ Rush Institute for Healthy Aging, Chicago, IL USA; ^2^ Rush Alzheimer's Disease Center, Rush University Medical Center, Chicago, IL USA

## Abstract

**Background:**

It is unclear whether inflammation, i.e., high interleukin‐6 (IL‐6) levels, and genetic risk, i.e., APOE ε4 allele, have a compounding effect on cognitive decline (CD).

**Method:**

We analyzed a subset of participants from the longitudinal cohort study, Chicago Health and Aging Project, comprising 1,120 biracial community‐dwelling older adults (60% African American & 62% women), and mean follow‐up=6.4 years. We ran adjusted mixed‐effects models of longitudinal cognitive decline and explored potential racial differences.

**Result:**

In ε4 carriers, higher serum IL‐6 was not associated with the rate of CD (β=‐0.0091 (SD=0.0165, p=0.5800). Conversely, in non‐ε4 carriers, compared to the lower tertile, those with the upper tertile of serum IL‐6 levels experienced significantly accelerated CD [β=‐0.0257 (SD=0.0084 p=,0.0023)]. These results did not differ between races.

**Conclusion:**

Even without the largest genetic risk factor for late‐onset AD/ADRD, elevated serum IL‐6 still accelerate the rate of CD. Hence, interventions ameliorating inflammation may prevent AD/ADRD. In contrast to the ε4 allele, as serum IL‐6 is a modifiable risk factor, these findings could implicate precision medicine, including AD/ADRD preventions and therapies to prevent AD/ADRD.